# Dietary sodium, table salt use, and specific depressive symptoms: cross-sectional associations in NHANES and an independent Chinese clinical cohort

**DOI:** 10.3389/fnut.2026.1838842

**Published:** 2026-06-15

**Authors:** Qilong Wang, Siheng Ma, Cong Cao, Xin Qi, Jianpeng Zhao, Dongmei Ma, Lei Zhang, Sha Liu, Yan He, Dongrong Zhao

**Affiliations:** 1 Department of Psychiatry, Gansu Provincial People’s Hospital, The First Clinical College of Gansu University of Chinese Medicine, Lanzhou, Gansu, China; 2 Department of Psychiatry, Xijing Hospital, The Fourth Military Medical University, Xi’an, Shaanxi, China

**Keywords:** Chinese clinical cohort, dietary sodium intake, NHANES, restricted cubic spline, specific depressive symptoms, table salt addition

## Abstract

**Background:**

The associations of dietary sodium intake and habitual table salt use with depressive symptoms may vary across symptom domains, but their relationships with specific depressive symptoms and potential non-linear patterns remain insufficiently understood. This study examined these associations in a US national sample and assessed whether directionally similar patterns could be observed in an independent Chinese clinical cohort.

**Methods:**

We analyzed data from 23,753 adults in the NHANES (2005–2023) and an independent Chinese clinical cohort of 700 participants. Multivariable regression models and restricted cubic spline (RCS) analyses were used to evaluate the associations of habitual table salt addition and dietary sodium intake with individual PHQ-9 depressive symptoms. Subgroup analyses and interaction tests stratified by age, gender, education, and hypertension status were performed to explore potential effect modification.

**Results:**

In NHANES, habitual table salt addition was associated with higher odds of eight depressive symptoms (ORs 1.24–1.37), including low self-esteem, depressed mood, anhedonia, fatigue, difficulty concentrating, psychomotor changes, appetite change, and sleep disturbance. Directionally similar but less consistent nominal associations were observed in the Chinese clinical cohort; however, symptom-level associations in this cohort did not remain statistically significant after multiple-testing correction. Restricted cubic spline analyses suggested non-linear associations between dietary sodium intake and the odds of depressive symptom presence, with the fitted spline curves reaching their nadirs at approximately 4.0–4.6 g/day of sodium in the study data. Subgroup analyses did not identify any interaction that remained statistically significant after multiple-testing correction.

**Conclusion:**

Habitual table salt addition was associated with multiple depressive symptoms in NHANES, with partially consistent patterns observed in the Chinese clinical cohort, particularly for somatic symptoms. Dietary sodium intake showed non-linear associations with the odds of depressive symptom presence. However, the nadir values observed in the spline analyses should not be interpreted as optimal, beneficial, safe, or recommended sodium intake targets, given the cross-sectional design and the limitations of dietary exposure assessment. These findings should be regarded as hypothesis-generating and support the need for prospective and interventional studies to clarify temporality, causality, and clinical relevance.

## Graphical Abstract

**Figure:**
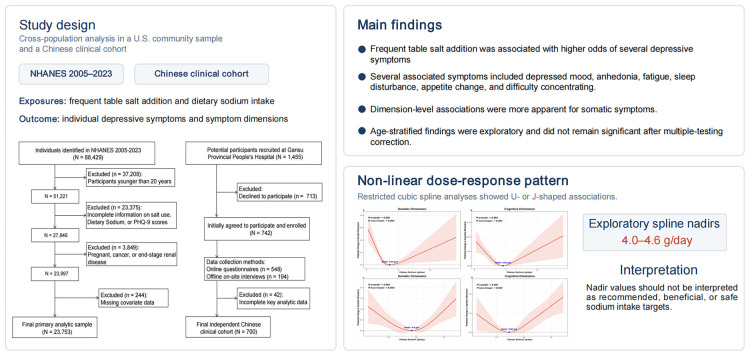
Overview of the study design and key findings.

## 1 Introduction

Depression is a leading cause of disability worldwide. Beyond persistent low mood, depression often includes anhedonia, low energy, and sleep problems—symptoms vary widely between patients (1, 2). The 2021 Global Burden of Disease (GBD) study estimates that depression affects about 332 million people, making it the second-leading cause of years lived with disability (3). Depression often coexists with chronic conditions like heart disease and diabetes (4, 5). This makes things worse for patients and strains healthcare systems. But depression is often missed early on, so many patients do not receive timely treatment (6). Finding ways to prevent depression and catch it early is key to reducing its impact on society (7).

Dietary patterns are potentially relevant correlates of mental health outcomes. Growing evidence suggests that overall diet quality and specific dietary factors are associated with depression and other mental health outcomes (8, 9). Data from the UK Biobank—a large-scale prospective cohort involving hundreds of thousands of participants—demonstrate that individuals who consistently add salt at the table have an approximately 37% higher risk of depression than those who never or rarely do so (10). In patients with primary aldosteronism, reducing daily salt intake from 9.1 g/day to 5.2 g/day was associated with improved depressive symptoms and overall mental well-being, suggesting a possible link between salt-related physiological regulation and mental health in selected clinical populations (11). When evaluating long-term salt exposure, the frequency of table salt addition serves as a practical behavioral marker. This approach offers distinct advantages, as it better captures stable dietary habits and is less prone to intraday fluctuations than short-term dietary recall. Existing work has estimated that table salt contributes roughly 6–20% of total sodium intake in Western diets (12). Using salting practices as an entry point may help capture habitual sodium-related exposure patterns relevant to health outcomes (13). Despite these findings, evidence on the associations between sodium-related exposures and specific depressive symptoms remains limited.

Most prior research has examined sodium-related exposures in relation to overall depression status or total depressive symptom scores (10, 14, 15). Although this approach facilitates comparisons across studies, it may overlook the heterogeneity of depressive symptoms. Different depressive symptoms may reflect partially distinct risk-factor profiles, biological correlates, and clinical implications (16, 17), and may therefore respond differently to sodium-related exposures. Current evidence on table salt addition and depression has largely come from Western population-based cohorts (10, 14), whereas evidence from Chinese populations remains limited despite their distinct dietary habits and typically higher salt intake (18, 19). This gap limits the broader application of findings across diverse populations.

In this study, we analyzed nationally representative NHANES data (2005–2023) and assessed whether directionally similar patterns could be observed in an independent Chinese clinical cohort from Gansu Provincial People’s Hospital. We evaluated how the frequency of table salt addition and dietary sodium intake were associated with individual PHQ-9 symptoms and symptom dimensions. Dose-response analyses were conducted to explore potential non-linear associations. The nadir values identified in the spline models were interpreted descriptively and were not intended to represent optimal or recommended intake targets.

## 2 Materials and methods

### 2.1 Participants

We obtained data from the National Health and Nutrition Examination Survey (NHANES). The NHANES, run by the National Center for Health Statistics (NCHS), is a cross-sectional survey that monitors the health and nutritional status of American adults and children. The survey uses multistage probability sampling to select participants from the non-institutionalized U.S. population. Data came from two sources: household interviews collected questionnaire data on demographics, socioeconomic status, and dietary habits; Mobile Examination Centres (MECs) performed physical exams, psychological assessments, and laboratory tests, including blood sampling. We combined nine NHANES cycles (2005–2023) for analysis.

**FIGURE 1:**
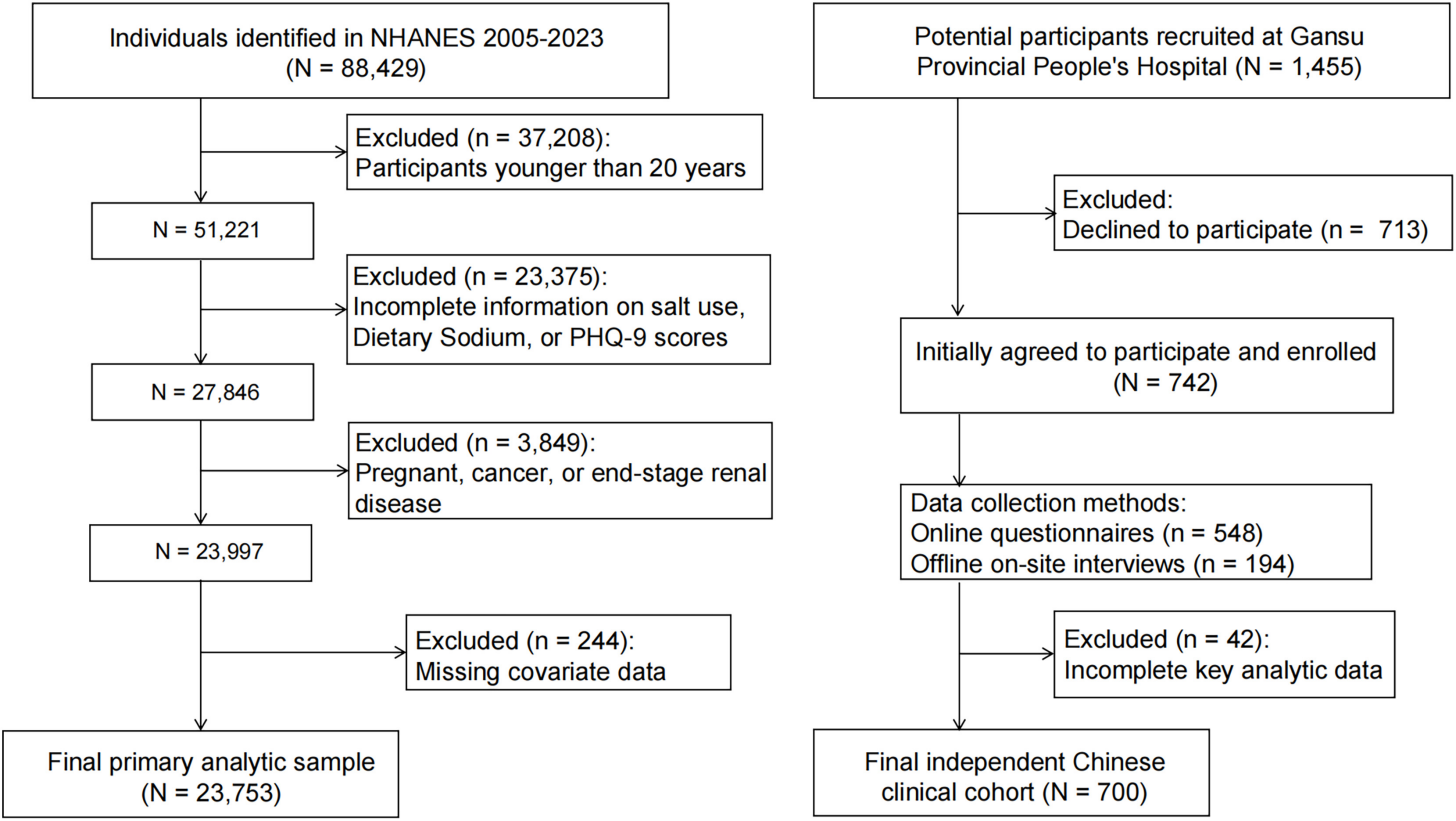
Flowchart of participants in this study.

We established a clinical cohort in the Department of Psychiatry at Gansu Provincial People’s Hospital from June to December 2025. A total of 1,455 potential participants aged ≥ 20 years were screened through outpatient and inpatient registration systems. To match the NHANES cohort, inclusion required age ≥ 20 years, and the same exclusion criteria were applied, including pregnancy, history of malignant tumors or cancer, and end-stage renal disease or renal failure. Among the screened individuals, 713 declined to participate, and 742 initially agreed to participate and were enrolled. Data were collected through online questionnaires (*n* = 548) and offline on-site interviews (*n* = 194). After excluding 42 participants with incomplete key analytic data, 700 participants were included in the final analysis. This study was approved by the Ethics Committee of Gansu Provincial People’s Hospital (Approval No. 2025-439). All participants consented to anonymous data use. The inclusion and exclusion process is illustrated in Figure 1.

### 2.2 Assessment of salt intake

This study employed table salt addition frequency and dietary sodium intake to reflect long-term salting habits and recent sodium consumption levels, respectively. Table salt addition frequency was used as a behavioral marker of discretionary salt use and salt preference, while 24-h dietary sodium intake was analyzed separately to capture recent dietary sodium intake. Table salt addition frequency was derived from the baseline questionnaire item: “How often do you add extra salt at the table when eating (excluding salt used in cooking)?” Participants were categorized into the rarely/occasionally and frequently groups to contrast non-frequent versus habitual frequent table salt addition and to maintain stable model estimation across cohorts; non-respondents were treated as missing data (20). Dietary sodium intake was calculated from 24-h dietary recalls and expressed as grams per day of sodium, rather than grams per day of salt (sodium chloride, NaCl), to avoid confusion between sodium and salt units. For NHANES participants who completed two recalls, one in person at the MEC and one by telephone 3–10 days later, the mean of the two recalls was used as the daily average intake; for those who completed only one recall, the first recorded value was used (21). The Gansu cohort followed the same 24-h dietary recall framework and grouping criteria as NHANES. Dietary sodium intake was calculated by matching reported foods and beverages with sodium values from the Chinese Food Composition Tables, summing sodium intake across all items, and converting mg/day to g/day of sodium.

### 2.3 Assessment of depressive symptoms and dimensions

This study employed the Patient Health Questionnaire-9 (PHQ-9) to assess depressive symptoms and their dimensions. The PHQ-9 comprises nine items: anhedonia, depressed mood, sleep disturbance, fatigue, appetite change, low self-esteem, difficulty concentrating, psychomotor changes, and suicidal ideation. Each item is scored from 0 to 3 according to symptom frequency. A score of ≥ 2 was defined as symptom presence, whereas scores of 0–1 were defined as absence of that specific symptom and served as the reference group in symptom-specific analyses. The denominator for each symptom-specific analysis was the full analytic sample in each cohort: 23,753 participants in NHANES and 700 participants in the Gansu cohort. Sleep disturbance, fatigue, appetite change, and psychomotor changes were summed as the somatic dimension, with a possible score range of 0–12. Anhedonia, depressed mood, low self-esteem, difficulty concentrating, and suicidal ideation were summed as the cognitive-emotional dimension, with a possible score range of 0–15. Higher scores indicated greater symptom burden within each dimension. Both cohorts used the same PHQ-9 coding and dimension scoring rules.

### 2.4 Assessment of covariates

We adjusted for multiple confounders, including demographic factors (gender, age, marital status, educational attainment, and household income poverty ratio [PIR]), lifestyle factors (smoking and alcohol use), body mass index (BMI), and self-reported chronic diseases (hypertension, diabetes mellitus, and cardiovascular disease). Within the NHANES cohort, smoking was defined as “never smoked” or “current smoker” based on interview data; alcohol use meant drinking more than 5 drinks daily in the past year. BMI was grouped as < 18.5, 18.5–24.9, 25.0–29.9, and ≥ 30.0 kg/m^2^. Chronic diseases were based on self-reported doctor diagnoses. The independent Chinese clinical cohort from Gansu Provincial People’s Hospital did not include race because all participants were Han Chinese; definitions, collection, and grouping criteria for other covariates were harmonized with NHANES where applicable. Total energy intake, overall diet quality, potassium intake, physical activity, antidepressant use, psychiatric history, and other medication use were not included in the main models because they were not consistently available or could not be reliably harmonized across the two cohorts. Sleep-related variables were not additionally adjusted for because sleep disturbance was one of the PHQ-9 symptom outcomes.

**TABLE 1:** Baseline characteristics, sodium-related exposures, and depressive symptoms in the NHANES and Gansu cohorts.

Characteristic	NHANES	Gansu Provincial People’s Hospital	Test statistic	*P*-value
Age (years), mean (SD)	45.0 (16.2)	52.5 (17.5)	*t* = 10.63	< 0.001
**Gender, n (%)**			**χ ^2^ = 17.11**	**< 0.001**
Male	12,038 (50.4)	297 (42.4)		
Female	11,715 (49.6)	403 (57.6)		
**Race, n (%)**			**/**	**/**
Mexican American	3,775 (9.1)	/		
Other Hispanic	2,203 (6.1)	/		
Non-Hispanic White	10,600 (66.6)	/		
Non-Hispanic Black	4,798 (10.6)	/		
Other Race	2,377 (7.7)	/		
**Education, n (%)**			**χ ^2^ = 28.18**	**< 0.001**
Less than high school	5,083 (13.7)	60 (8.6)		
High school or equivalent	5,581 (24.3)	140 (20.0)		
College or above	13,089 (62.0)	500 (71.4)		
**Marital status, n (%)**			**χ ^2^ = 87.46**	**< 0.001**
Married or living with a partner	14,069 (62.3)	377 (53.9)		
Never married	3,395 (13.7)	183 (26.1)		
Widowed/divorced/separated	6,289 (24.0)	140 (20.0)		
**PIR, n (%)**			**χ ^2^ = 206.68**	**< 0.001**
<130%	7,176 (21.6)	149 (21.3)		
130–349%	8,849 (34.9)	415 (59.3)		
≥ 350%	7,728 (43.6)	136 (19.4)		
**BMI, n (%)**			**χ ^2^ = 129.62**	**< 0.001**
<18.5	353 (1.4)	11 (1.6)		
18.5–24.9	6,547 (28.6)	311 (44.4)		
25.0–29.9	7,798 (32.9)	253 (36.1)		
≥ 30.0	9,055 (37.0)	125 (17.9)		
**Smoking status, n (%)**			**χ ^2^ = 0.79**	**0.391**
Non-smoker	18,453 (78.7)	561 (80.1)		
Smoker	5,300 (21.3)	139 (19.9)		
**Alcohol drinking status, n (%)**			**χ ^2^ = 5.88**	**0.019**
No excessive alcohol	19,674 (84.0)	612 (87.4)		
Excessive alcohol	4,079 (16.0)	88 (12.6)		
**Hypertension, n (%)**			**χ ^2^ = 12.34**	**< 0.001**
No	16,471 (72.9)	468 (66.9)		
Yes	7,282 (27.1)	232 (33.1)		
**Diabetes, n (%)**			**χ ^2^ = 19.56**	**< 0.001**
No	21,253 (92.1)	612 (87.4)		
Yes	2,500 (7.9)	88 (12.6)		
**CVD, n (%)**			**χ ^2^ = 22.80**	**< 0.001**
No	21,850 (93.7)	624 (89.1)		
Yes	1,903 (6.3)	76 (10.9)		
Dietary sodium (g/day), mean (SD)	3.5 (1.5)	4.5 (1.7)	*t* = 14.42	< 0.001
**Table salt use, n (%)**			**χ ^2^ = 9.92**	**0.002**
Rarely/Occasionally	18,651 (79.1)	588 (84.0)		
Often	5,102 (20.9)	112 (16.0)		
Depressive symptoms, n (%)				
Anhedonia	1,917 (7.1)	60 (8.6)	χ^2^ = 2.15	0.150
Depressed mood	1,735 (6.3)	54 (7.7)	χ^2^ = 2.15	0.151
Sleep disturbance	3,651 (15.3)	139 (19.9)	χ^2^ = 10.91	0.001
Fatigue	3,775 (15.1)	145 (20.7)	χ^2^ = 16.73	< 0.001
Appetite change	2,221 (8.4)	86 (12.3)	χ^2^ = 13.27	< 0.001
Low self-esteem	1,376 (5.4)	57 (8.1)	χ^2^ = 9.74	0.003
Concentration problems	1,478 (6.0)	81 (11.6)	χ^2^ = 36.11	< 0.001
Psychomotor changes	868 (3.2)	18 (2.6)	χ^2^ = 0.85	0.362
Suicidal ideation	239 (0.9)	11 (1.6)	χ^2^ = 3.31	0.078

SD, standard deviation; BMI, body mass index; PIR, poverty-income ratio; CVD, cardiovascular disease.

### 2.5 Statistical analyses

Given that NHANES employs a stratified, multistage, cluster probability sampling design, we incorporated sample weights, strata, and primary sampling units (PSUs) in NHANES descriptive analyses, regression models, and subgroup interaction analyses to obtain estimates representative of the US general population. To consolidate dietary data from nine survey cycles spanning 2005–2023, we followed NCHS guidelines to construct composite sample weights suitable for multi-cycle pooled data. For missing data, we used multiple imputation by chained equations (MICE) to create a complete dataset. Continuous variables were presented as weighted means ± SD, and categorical variables were presented as unweighted counts with weighted percentages. Between-cohort comparisons for variables in Table 1 were conducted using survey-weighted *t*-tests for continuous variables and survey-adjusted χ^2^ tests for categorical variables, incorporating the NHANES survey design. These comparisons were used to describe baseline differences between the NHANES and Gansu cohorts. We used weighted logistic regression for binary outcomes and weighted linear regression for continuous scores in NHANES, and corresponding unweighted models in the Gansu cohort because it had no complex sampling design. We built three models: Model 1 was unadjusted; Model 2 was adjusted for age, gender, marital status, education, and PIR; and Model 3 further adjusted for BMI, smoking, alcohol use, and history of CVD, diabetes, and hypertension. The dose-response relationship between dietary sodium intake and depressive phenotypes was modeled using RCS. For NHANES, weighted RCS models were used; for the Gansu cohort, unweighted RCS models were used. For outcomes exhibiting significant non-linearity, the nadir point, defined as the sodium intake level corresponding to the lowest fitted value within the prediction curve, was identified via prediction curves and employed as a descriptive reference point for calculating odds ratios (ORs) or predicted changes. These nadir values were interpreted descriptively and were not intended to represent optimal or recommended intake targets. For analyses involving multiple PHQ-9 symptom outcomes, depressive symptom dimensions, RCS non-linearity tests, or subgroup interaction tests, both uncorrected *P*-values and multiple-testing corrected *P*-values using Bonferroni and Sidak methods were reported in Supplementary Tables 1–4.

We analyzed the data using R statistical software version 4.4.2 (R Foundation for Statistical Computing, Vienna, Austria). A two-sided *P* < 0.05 was considered statistically significant.

### 2.6 Subgroup and interaction analyses

We conducted subgroup analyses stratified by age, gender, education, and hypertension status. For NHANES, survey-weighted logistic regression models were used; for the Gansu cohort, unweighted logistic regression models were used. Interaction terms between table salt addition and each subgroup factor were added to assess potential effect modification. Wald tests were used to obtain interaction *P*-values. Parallel analyses were conducted in the independent Chinese clinical cohort using the same definitions and similar models where applicable.

## 3 Results

### 3.1 Description of study participants

We included 23,753 participants from NHANES (2005–2023) and 700 participants from Gansu Provincial People’s Hospital. Baseline characteristics are shown in Table 1. In NHANES, the mean age was 45.0 years, and men and women were nearly equally represented. College education or above was reported by 62.0% of participants. The prevalence of obesity (BMI ≥ 30 kg/m^2^), smoking, and heavy drinking was 37.0, 21.3, and 16.0%, respectively. Hypertension was present in 27.1% of participants, and cardiovascular disease was present in 6.3%. Dietary sodium intake was 3.5 ± 1.5 g/day of sodium, and 20.9% of participants reported frequently adding table salt to food. The prevalence of individual depressive symptoms ranged from 0.9 to 15.3%, with sleep disturbance (15.3%) and fatigue (15.1%) being the most common, and suicidal ideation (0.9%) being the least common.

Compared with the NHANES cohort, participants in the Gansu cohort were older, with a mean age of 52.5 years, and a higher proportion were women (57.6%). The Gansu cohort also had a higher prevalence of chronic diseases, including hypertension (33.1%) and cardiovascular disease (10.9%). By contrast, the prevalence of obesity was lower in the Gansu cohort (17.9%), while smoking and heavy drinking were reported by 19.9% and 12.6% of participants, respectively. In the Gansu cohort, dietary sodium intake was 4.5 ± 1.7 g/day of sodium, and 16.0% of participants reported frequently adding table salt to food. Fatigue (20.7%) and sleep disturbance (19.9%) were the most common depressive symptoms, whereas suicidal ideation was the least common (1.6%). Between-cohort comparisons showed significant differences in age, gender, education, marital status, PIR, BMI, alcohol drinking status, hypertension, diabetes, CVD, dietary sodium intake, table salt use, and several depressive symptoms, including sleep disturbance, fatigue, appetite change, low self-esteem, and concentration problems (all *P* < 0.05; Table 1).

Data are presented as unweighted numbers of participants with weighted percentages for NHANES and as numbers with percentages for the Gansu cohort, or as mean (SD) where applicable. NHANES estimates were weighted to be nationally representative. Race was not reported for the Gansu cohort because all participants were Han Chinese.

Between-cohort comparisons were conducted using survey-weighted *t*-tests for continuous variables and survey-adjusted χ^2^ tests for categorical variables, incorporating the NHANES survey design where applicable. These comparisons were intended to describe baseline differences between cohorts and should be interpreted cautiously because the cohorts were derived from different source populations.

### 3.2 Associations of table salt use and dietary sodium intake with specific depressive symptoms

Table 2 shows the association between table salt addition frequency and nine depressive symptoms. In Model 3, frequent salt addition was associated with higher odds of most symptoms in NHANES and showed directionally similar nominal associations for several symptoms in the Gansu cohort. In NHANES, adding salt frequently, compared with rarely or occasionally, was associated with higher odds of eight symptoms: low self-esteem (OR = 1.37, 95% CI: 1.13–1.67, *P* = 0.002), depressed mood (OR = 1.31, 95% CI: 1.08–1.60, *P* = 0.007), anhedonia (OR = 1.29, *P* = 0.001), fatigue (OR = 1.28, *P* < 0.001), difficulty concentrating (OR = 1.28, *P* = 0.008), psychomotor changes (OR = 1.27, *P* = 0.031), appetite change (OR = 1.27, *P* = 0.003), and sleep disturbance (OR = 1.24, *P* < 0.001). Suicidal ideation showed no significant association (*P* = 0.207).

**TABLE 2:** Table salt use and depression symptoms: results from weighted NHANES and Chinese cohorts.

Specific depressive symptom	NHANES			Gansu Provincial People’s Hospital		
	Model 1	Model 2	Model 3	Model 1	Model 2	Model 3
	OR (95% CI) *P*-value	OR (95% CI) *P*-value	OR (95% CI) *P*-value	OR (95% CI) *P*-value	OR (95% CI) *P*-value	OR (95% CI) *P*-value
Anhedonia	1.45 (1.25, 1.68) < 0.001	1.34 (1.15, 1.56) < 0.001	1.29 (1.11, 1.50) 0.001	1.87 (0.97, 3.41) 0.050	1.83 (0.93, 3.41) 0.067	1.97 (1.00, 3.71) 0.042
Depressed mood	1.47 (1.21, 1.79) < 0.001	1.36 (1.12, 1.65) 0.002	1.31 (1.08, 1.60) 0.007	2.41 (1.26, 4.43) 0.006	2.44 (1.25, 4.60) 0.007	2.37 (1.19, 4.56) 0.011
Sleep disturbance	1.32 (1.17, 1.49) < 0.001	1.28 (1.14, 1.44) < 0.001	1.24 (1.10, 1.39) < 0.001	1.80 (1.12, 2.83) 0.013	1.91 (1.17, 3.10) 0.009	1.75 (1.05, 2.87) 0.029
Fatigue	1.35 (1.21, 1.52) < 0.001	1.31 (1.17, 1.47) < 0.001	1.28 (1.14, 1.44) < 0.001	1.78 (1.12, 2.78) 0.014	1.82 (1.12, 2.92) 0.014	1.85 (1.13, 2.99) 0.013
Appetite change	1.35 (1.16, 1.56) < 0.001	1.30 (1.12, 1.52) < 0.001	1.27 (1.09, 1.48) 0.003	1.86 (1.06, 3.14) 0.025	2.03 (1.14, 3.50) 0.013	1.98 (1.11, 3.45) 0.018
Low self-esteem	1.48 (1.22, 1.80) < 0.001	1.42 (1.17, 1.73) < 0.001	1.37 (1.13, 1.67) 0.002	1.62 (0.81, 3.05) 0.147	1.75 (0.86, 3.37) 0.105	1.67 (0.81, 3.29) 0.149
Concentration problems	1.37 (1.15, 1.64) < 0.001	1.31 (1.10, 1.56) 0.003	1.28 (1.07, 1.54) 0.008	2.03 (1.16, 3.45) 0.011	2.11 (1.17, 3.71) 0.011	2.02 (1.11, 3.64) 0.019
Psychomotor changes	1.49 (1.21, 1.85) < 0.001	1.34 (1.07, 1.66) 0.009	1.27 (1.02, 1.59) 0.031	/	/	/
Suicidal ideation	1.60 (0.97, 2.62) 0.064	1.42 (0.84, 2.40) 0.190	1.39 (0.83, 2.33) 0.207	0.52 (0.03, 2.76) 0.536	0.48 (0.02, 2.92) 0.506	0.33 (0.01, 2.78) 0.388

Model 1: unadjusted. Model 2: adjusted for age, gender, marital status, education, and PIR. Model 3: Model 2 + BMI, smoking status, alcohol drinking status, CVD, diabetes, and hypertension. BMI, body mass index; PIR, poverty-income ratio; CVD, cardiovascular disease; OR, odds ratio; CI, confidence interval.

In the Gansu cohort, numerically larger estimates were observed for several symptoms, although these associations should be interpreted cautiously because they were nominally significant before multiple-testing correction. Frequent table salt addition showed nominal associations with higher odds of depressed mood (OR = 2.37, 95% CI: 1.19–4.56, *P* = 0.011), difficulty concentrating (OR = 2.02, 95% CI: 1.11–3.64, *P* = 0.019), appetite change (OR = 1.98, 95% CI: 1.11–3.45, *P* = 0.018), anhedonia (OR = 1.97, 95% CI: 1.00–3.71, *P* = 0.042), fatigue (OR = 1.85, 95% CI: 1.13–2.99, *P* = 0.013), and sleep disturbance (OR = 1.75, 95% CI: 1.05–2.87, *P* = 0.029). Low self-esteem (*P* = 0.149) and suicidal ideation (*P* = 0.388) were not statistically significant. Psychomotor changes were excluded from the table salt addition regression analysis because of sparse outcomes and model instability.

**FIGURE 2:**
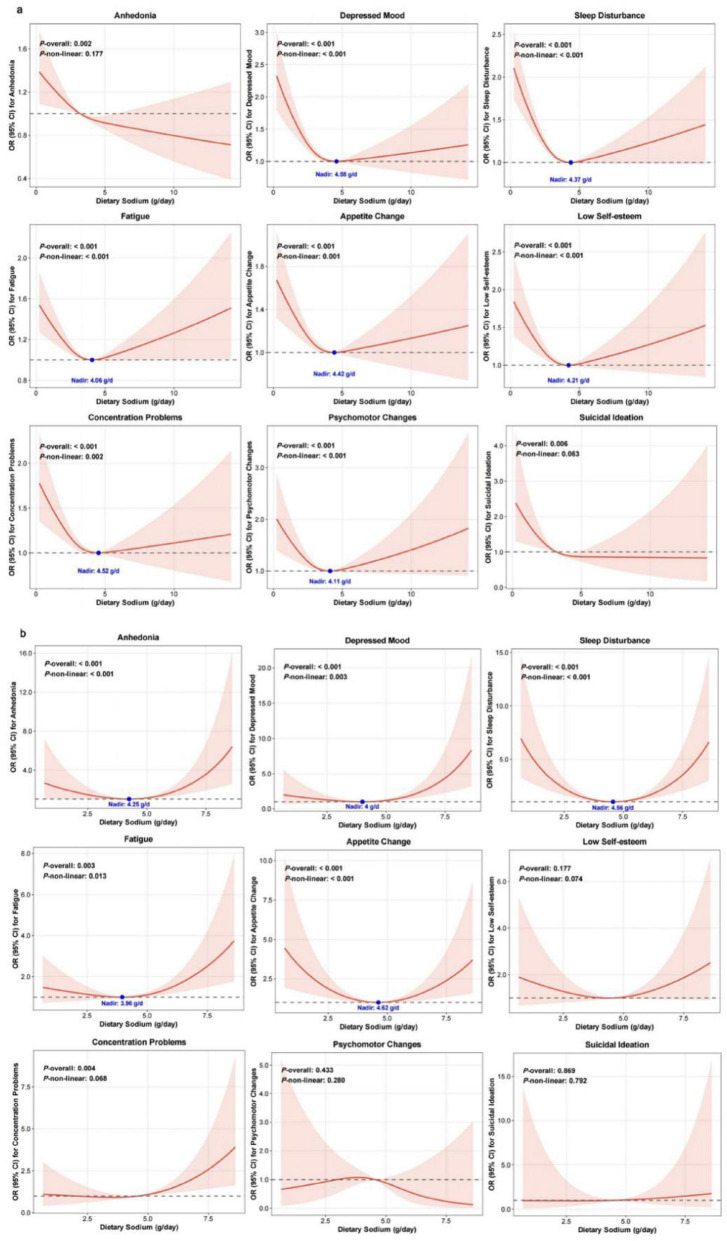
Non-linear associations between dietary sodium intake (g/day of sodium) and nine depressive symptoms. **(a)** Weighted results from NHANES, and **(b)** results from the Gansu cohort. Models were adjusted for covariates. *P*-values for non-linearity are shown. Shaded areas indicate 95% confidence intervals.

Figure 2 shows the dose-response relationships between dietary sodium intake and nine depressive symptoms using restricted cubic spline models. In NHANES, dietary sodium intake showed non-linear associations with seven symptoms (all *P*_*non–linear*_ < 0.05). Fatigue (nadir 4.06 g/day of sodium), psychomotor changes (4.11 g/day of sodium), low self-esteem (4.21 g/day of sodium), sleep disturbance (4.37 g/day of sodium), and appetite change (4.42 g/day of sodium) showed U-shaped patterns, with higher fitted odds observed at intake levels above or below the nadir range. Difficulty concentrating (nadir 4.52 g/day of sodium) and depressed mood (4.58 g/day of sodium) showed J-shaped patterns. Anhedonia (*P*_*non–linear*_ = 0.177) and suicidal ideation (*P*_*non–linear*_ = 0.063) did not show statistically significant non-linear associations.

In Gansu, five symptoms showed non-linear associations (*P*_*non–linear*_ < 0.05). Fatigue (nadir 3.96 g/day of sodium), sleep disturbance (4.56 g/day of sodium), appetite change (4.62 g/day of sodium), and anhedonia (4.25 g/day of sodium) showed U-shaped patterns. Depressed mood showed a J-shaped pattern (nadir 4.00 g/day of sodium). Difficulty concentrating (*P*_*non–linear*_ = 0.068), low self-esteem (*P*_*non–linear*_ = 0.074), psychomotor changes (*P*_*non–linear*_ = 0.280), and suicidal ideation (*P*_*non–linear*_ = 0.792) were not statistically significant. Although the nadir values for several symptom curves clustered around 4.0–4.6 g/day of sodium, these findings should be interpreted descriptively rather than as evidence of optimal, beneficial, safe, or recommended sodium intake targets.

### 3.3 Associations of table salt use and dietary sodium intake with depressive symptom dimensions

Table 3 presents linear regression results for table salt addition frequency and depression dimension scores. In NHANES (Model 3), frequent salt addition was associated with higher scores on both dimensions. Compared with rare/occasional use, somatic symptom scores increased by 0.24 points (β = 0.24, 95% CI: 0.14–0.33, *P* < 0.001), and cognitive symptom scores increased by 0.23 points (β = 0.23, 95% CI: 0.11–0.34, *P* < 0.001). In the independent Chinese clinical cohort from Gansu, the same model showed a similar direction: somatic symptom scores increased by 0.49 points (β = 0.49, 95% CI: 0.02–0.96, *P* = 0.041), whereas cognitive symptom scores increased by 0.34 points but did not reach statistical significance (95% CI: -0.17–0.84, *P* = 0.20). Given the possible score ranges of 0–12 for the somatic dimension and 0–15 for the cognitive-emotional dimension, these coefficients represent relatively small absolute differences in dimension scores; therefore, their practical significance should be interpreted cautiously.

**TABLE 3:** Table salt use and depression dimensions: linear regression in weighted NHANES and Chinese cohorts.

Depressive symptom dimension	NHANES			Gansu Provincial People’s Hospital		
	Model 1	Model 2	Model 3	Model 1	Model 2	Model 3
	β (95% CI) *P*-value	β (95% CI) *P*-value	β (95% CI) *P*-value	β (95% CI) *P*-value	β (95% CI) *P*-value	β (95% CI) *P*-value
Somatic dimension	0.30 (0.20, 0.40) < 0.001	0.27 (0.18, 0.37) < 0.001	0.24 (0.14, 0.33) < 0.001	0.55 (0.06, 1.04) 0.028	0.58 (0.11, 1.05) 0.016	0.49 (0.02, 0.96) 0.041
Cognitive dimension	0.31 (0.19, 0.43) < 0.001	0.27 (0.15, 0.38) < 0.001	0.23 (0.11, 0.34) < 0.001	0.33 (-0.19, 0.86) 0.215	0.38 (-0.13, 0.88) 0.148	0.34 (-0.17, 0.84) 0.192

Model 1: unadjusted. Model 2: adjusted for age, gender, marital status, education, and PIR. Model 3: Model 2 + BMI, smoking status, alcohol drinking status, CVD, diabetes, and hypertension. BMI, body mass index; PIR, poverty-income ratio; CVD, cardiovascular disease; CI, confidence interval.

**FIGURE 3:**
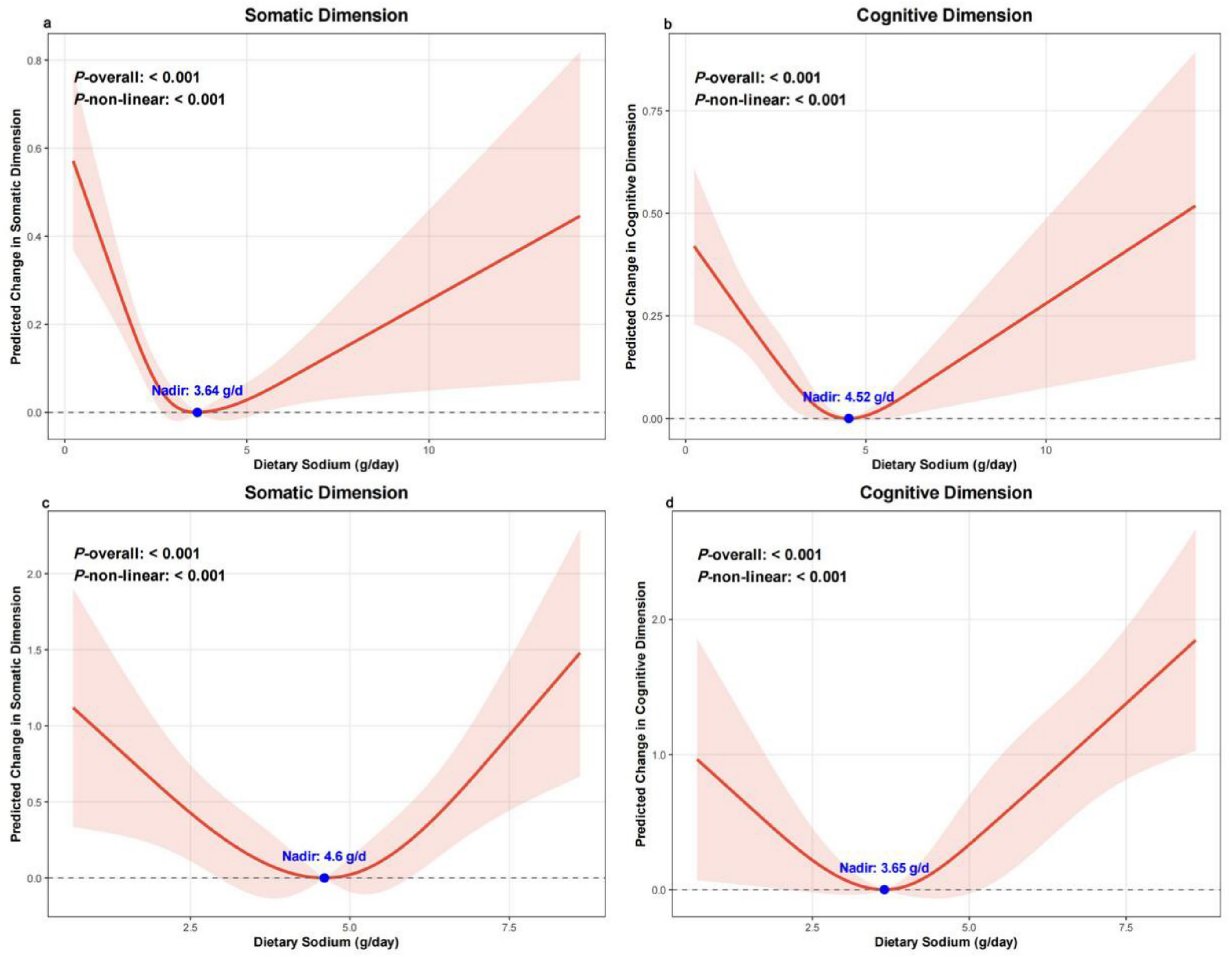
Non-linear associations between dietary sodium intake (g/day of sodium) and somatic and cognitive depressive symptom dimensions. **(a,b)** NHANES results for the somatic and cognitive dimensions, respectively, and **(c,d)** corresponding results from the Gansu cohort. Models were adjusted for covariates. All curves showed significant non-linearity (*P*_*non–linear*_ < 0.001). Shaded areas indicate 95% confidence intervals.

Figure 3 shows non-linear dose-response patterns between dietary sodium intake and depression dimension scores from RCS analyses. After adjustment for covariates, dietary sodium intake showed significant non-linear associations with both somatic and cognitive dimensions in both cohorts (all *P*_*non–linear*_ < 0.001). In NHANES (Figures 3a,b), the somatic dimension showed a U-shaped pattern (nadir 3.64 g/day of sodium), whereas the cognitive dimension showed a J-shaped pattern (nadir 4.52 g/day of sodium). In Gansu (Figures 3c,d), both dimensions showed U-shaped patterns, with nadir values of 4.60 g/day of sodium for the somatic dimension and 3.65 g/day of sodium for the cognitive dimension. These nadir values were interpreted descriptively and should not be regarded as optimal or recommended intake levels.

### 3.4 Subgroup analyses of the associations between table salt use and specific depressive symptoms

**FIGURE 4:**
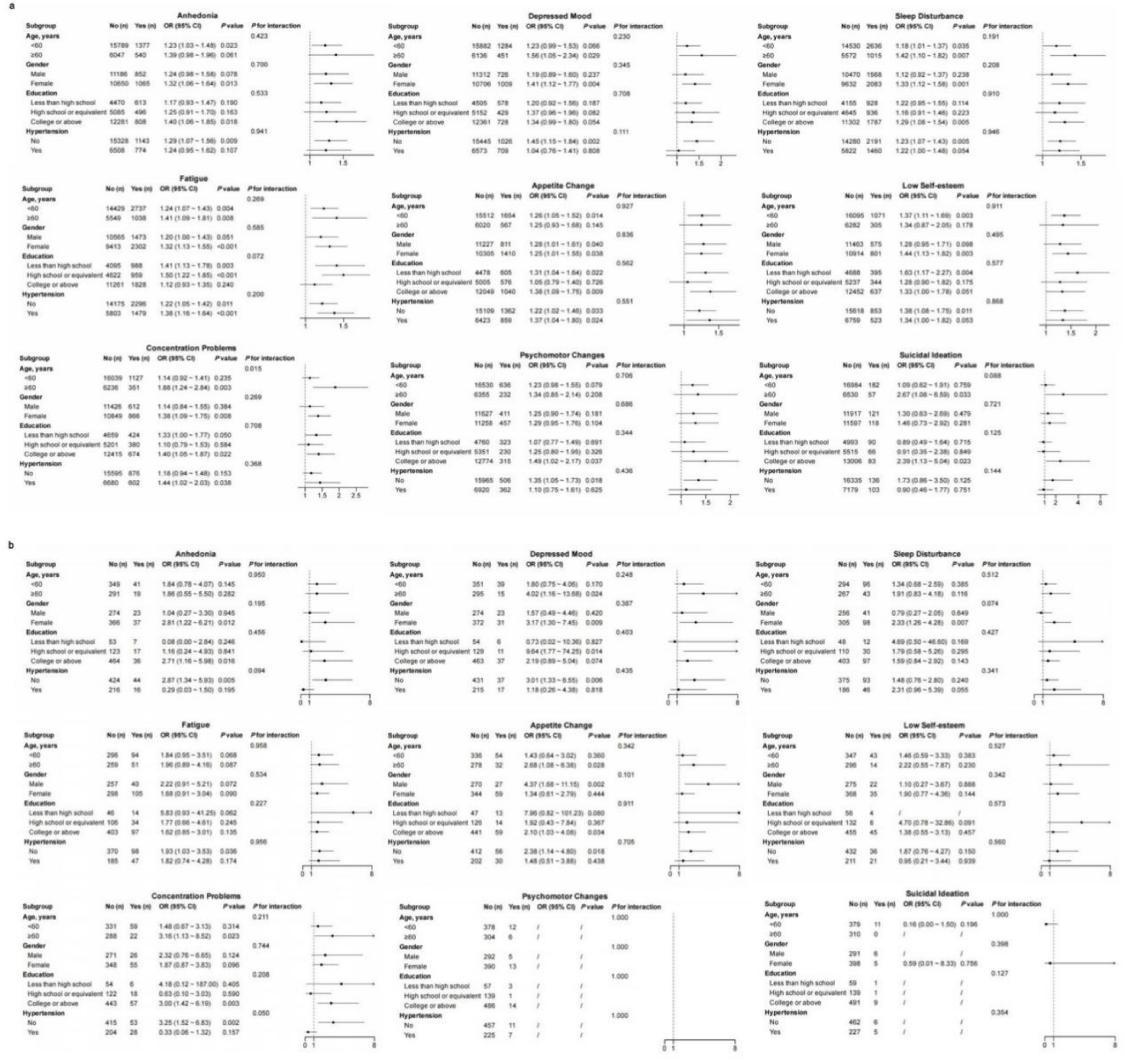
Subgroup analyses of table salt use and nine depression symptoms. **(a)** NHANES results. **(b)** Gansu results. Stratified by age, gender, education, and hypertension. Data: ORs and 95% CIs. Slashes (/) indicate subgroups with insufficient data for estimation.

Figure 4 shows subgroup analyses for table salt addition and nine depressive symptoms. In NHANES, associations between frequent salt addition and most symptoms were generally stable across age, gender, education, and hypertension status. Difficulty concentrating showed a nominal age interaction before multiple-testing correction (uncorrected *P* for interaction = 0.015), but this interaction did not remain statistically significant after Bonferroni or Sidak correction. In adults aged ≥ 60 years, frequent salt addition was associated with higher odds of difficulty concentrating (OR = 1.68, 95% CI: 1.24–2.84, *P* = 0.003); in those aged < 60 years, the association was not statistically significant (OR = 1.14, 95% CI: 0.92–1.41, *P* = 0.235). The Gansu cohort showed generally consistent directions, and no subgroup interaction remained statistically significant after multiple-testing correction.

### 3.5 Multiple-testing correction results

Multiple-testing correction results are summarized in Supplementary Tables 1–4. For the associations between table salt addition and individual depressive symptoms, several associations remained statistically significant after Bonferroni and Sidak correction in NHANES, including anhedonia, sleep disturbance, fatigue, appetite change, and low self-esteem, with corrected *P*-values < 0.05. In contrast, none of the symptom-level associations in the Gansu cohort remained statistically significant after correction. For depressive symptom dimensions, the associations of table salt addition with both somatic and cognitive dimensions remained statistically significant after correction in NHANES, whereas the association with the somatic dimension in the Gansu cohort was statistically significant before correction but did not remain significant after multiple-testing correction. For the RCS analyses, non-linearity remained statistically significant after correction for seven individual depressive symptoms in NHANES and four individual depressive symptoms in the Gansu cohort, and the non-linearity tests for both depressive symptom dimensions remained statistically significant after correction in both cohorts. For subgroup interaction tests, no interaction remained statistically significant after Bonferroni or Sidak correction within each cohort and subgroup factor, with corrected *P* > 0.05.

## 4 Discussion

We examined table salt addition behavior and dietary sodium intake in relation to specific depressive symptoms in NHANES and an independent Chinese clinical cohort. In NHANES, frequent table salt addition was associated with higher odds of several depressive symptoms, while the Chinese clinical cohort showed directionally similar but less robust nominal associations, with generally larger but less precise estimates. Restricted cubic spline analyses suggested non-linear associations between dietary sodium intake and several depressive symptoms, with U-shaped or J-shaped patterns observed for multiple outcomes. The nadir values of several fitted curves clustered around approximately 4.0–4.6 g/day of sodium within the study data; however, these values should be interpreted descriptively rather than as optimal or recommended intake targets. In addition, frequent table salt addition was associated with higher somatic symptom scores in both cohorts. Overall, these findings extend the existing literature by characterizing sodium-related exposures in relation to depressive symptoms at the symptom level, while remaining exploratory given the cross-sectional design.

### 4.1 Symptom-specific associations and non-linear dose-response patterns

Previous epidemiological studies have linked salt-related exposures to depression. An earlier analysis of the US general population reported associations between sodium-related exposures, including added salt, and depression, with patterns differing by exposure type and sex (15). Evidence from the UK Biobank further supported this association, showing that individuals who always added salt to food had a higher risk of depression than those who never or rarely did so (10). Another study combining observational analysis with Mendelian randomization also supported a potential association between frequent salt addition and major depressive disorder (22). However, these studies primarily used total depression scores and therefore may have overlooked symptom-level heterogeneity. In the present study, we extended this literature by examining specific depressive symptoms and found non-linear associations between dietary sodium intake and several symptom domains, with U-shaped or J-shaped patterns observed in both cohorts. The nadir values of several fitted curves clustered around approximately 4.0–4.6 g/day of sodium; however, these values should be interpreted descriptively rather than as optimal or recommended intake targets. Our findings are also broadly consistent with animal evidence. For example, Lu et al. reported that high-salt diets induced depression-like behaviors in mice, at least partly through increased IL-17A production and γδT17 cell induction (23). Although animal and human findings are not directly comparable, these findings provide experimental support for a possible link between high salt exposure, immune activation, and depressive phenotypes.

### 4.2 Cross-national comparison and subgroup heterogeneity

This study found broadly similar association patterns in a US community-based national sample and an independent Chinese clinical cohort. However, because the Chinese cohort was derived from a psychiatric clinical setting rather than a community-based population, it should be interpreted as an independent clinical comparison cohort rather than a formal validation sample for NHANES. In both cohorts, habitual table salt addition was associated with higher odds of several depressive symptoms, including depressed mood, anhedonia, sleep disturbance, fatigue, appetite changes, and difficulty concentrating. Symptom dimension analysis further supported these findings, with positive associations between habitual table salt addition and higher somatic symptom scores in both cohorts (NHANES: β = 0.24; Chinese clinical cohort: β = 0.49). The somatic dimension—encompassing sleep, fatigue, appetite, and psychomotor function—showed the most consistent association. However, the cognitive dimension did not reach statistical significance in the Chinese cohort. This discrepancy may reflect differences in sample composition or limited statistical power in the clinical cohort.

Effect estimates were generally larger in the Chinese clinical cohort than in the US community-based sample (e.g., depressed mood: OR = 2.37 vs. 1.31). This difference may partly reflect differences in source population, clinical characteristics, baseline symptom burden, comorbidity profiles, and dietary context. Because the Chinese cohort was recruited from a psychiatric clinical setting, participants may have had greater symptom burden and more homogeneous clinical characteristics than the general NHANES population. In addition, China’s generally high dietary salt burden may have contributed to a different exposure distribution. Therefore, differences in effect estimates between the two cohorts should be interpreted cautiously and should not be taken as evidence of direct comparability between the two populations.

The significant baseline differences shown in Table 1 should also be considered when interpreting the subsequent statistical analyses. The two cohorts differed in age distribution, gender composition, socioeconomic status, BMI, alcohol drinking status, cardiometabolic comorbidities, dietary sodium intake, table salt addition frequency, and several depressive symptoms. Although the multivariable models adjusted for major covariates, these baseline differences and other unmeasured cohort-specific factors may still have influenced the regression estimates in Tables 2, 3, the RCS-based dose-response curves in Figures 2, 3, and the subgroup patterns shown in Figure 4. Therefore, the findings from the two cohorts should be interpreted as cohort-specific associations rather than directly comparable effect sizes.

Age-stratified analyses suggested a nominally stronger association between frequent table salt addition and difficulty concentrating among individuals aged ≥ 60 years before multiple-testing correction. However, this interaction did not remain statistically significant after Bonferroni or Sidak correction; therefore, this finding should be interpreted cautiously. Although older adults may be more vulnerable to electrolyte fluctuations, low-grade inflammation, and altered vascular reactivity, the present results do not establish age-specific effects of table salt addition. Prior research has linked high salt exposure to blood-brain barrier disruption, neurovascular dysfunction, and cerebral small vessel disease (24–26). Thus, attention-related symptoms among older adults may warrant further investigation in future longitudinal studies, but this interpretation remains exploratory.

### 4.3 Potential mechanisms

After adjustment for multiple confounders, table salt addition remained associated with several depressive symptom outcomes. The observed associations appeared to vary across symptom domains, particularly for somatic symptoms such as fatigue, sleep disturbance, and appetite change. However, these findings should not be interpreted as evidence of causal effects. Instead, the symptom-specific patterns may provide hypotheses for future mechanistic, longitudinal, and interventional studies.

We observed associations between table salt addition and core depressive phenotypes (low mood, anhedonia). Salt craving involves more than taste—it engages the mesolimbic reward pathway. Hurley et al. (27) noted that the nucleus accumbens and its dopaminergic projections drive salt craving and reward. Ferraris et al. (28) found that liking of salt was associated with higher depression, anxiety, and stress scores. During depressive states, the reward system may shift how it encodes stimulus salience. Some individuals might seek stronger gustatory cues for temporary pleasure, which may be related to more frequent salt addition at meals.

We also observed an association between table salt addition and fatigue. Animal evidence suggests that high-salt diets may induce depressive-like behaviors through IL-17A-related immune pathways (23). In addition, high-salt exposure has been linked to neurovascular and cognitive dysfunction through gut-initiated TH17 responses (26). In population studies, peripheral inflammatory markers (e.g., neutrophil-to-lymphocyte ratio, NLR) correlate with specific depressive symptoms, including fatigue (29). Inflammatory signals can reach the brain through humoral routes, neurotransmission, and immune channels, triggering microglial activation and disrupting neurotransmitter balance. Inflammation activates indoleamine 2,3-dioxygenase (IDO), shifting the kynurenine pathway and causing neuroactive metabolite imbalances. This process links closely to “sickness behavior”—characterized by fatigue, anhedonia, and psychomotor retardation (30, 31). Our somatic symptom findings may reflect the connection between peripheral inflammatory burden and central functional changes.

The age-stratified pattern observed in NHANES should be interpreted cautiously. Frequent table salt addition showed a nominally stronger association with difficulty concentrating among adults aged ≥ 60 years before multiple-testing correction, but this interaction did not remain statistically significant after Bonferroni or Sidak correction. Nevertheless, this exploratory pattern may raise the hypothesis that vascular mechanisms are relevant to sodium-related cognitive symptoms. Previous studies have linked high salt intake to impaired endothelial function and blood pressure-related changes. Schneider et al. (11) found that salt restriction improved both blood pressure and mental health scales. A randomized crossover trial showed that high salt intake rapidly suppressed flow-mediated dilation (FMD), even in the absence of an immediate increase in blood pressure (32). Other studies have linked higher salt intake to a greater burden of cerebral small vessel disease on neuroimaging (25), and this association may be partly independent of blood pressure (33). From an endocrine perspective, Murck et al. (34) discussed mineralocorticoid receptor-related markers, including blood pressure and electrolytes, in relation to major depression outcomes, suggesting a possible role of neuroendocrine regulation. Reviews have also suggested that mineralocorticoid receptors help regulate basal HPA axis activity and stress appraisal (35). Experimental evidence further indicates that high salt intake may activate the HPA axis, amplify stress responses, and increase glucocorticoid exposure (36). Potential sodium-related mechanisms may also involve very low sodium intake. Strict sodium restriction can trigger compensatory activation of the renin-angiotensin-aldosterone system (RAAS), with increases in renin, aldosterone, and catecholamines (37). Such neuroendocrine changes have been linked to anxiety-related processes, and HPA axis dysregulation may also be involved in affective symptoms (38). Taken together, neurovascular and endocrine pathways may provide a plausible biological framework for the observed non-linear associations between dietary sodium intake and depressive symptoms, although these mechanisms remain speculative in the context of the present cross-sectional study.

### 4.4 Interpretation of the observed non-linear associations

The observed non-linear associations should be interpreted cautiously. In the present study, several fitted spline curves showed nadir values around 4.0–4.6 g/day of sodium. These nadir values represent exploratory estimates of the sodium intake levels corresponding to the lowest fitted odds or predicted scores within the observed data, rather than optimal, beneficial, safe, or recommended sodium intake targets. Importantly, these observational findings were not designed to challenge or replace guideline-based sodium reduction recommendations, which are primarily based on blood pressure and cardiovascular outcomes (39, 40). Given the cross-sectional design, reliance on 24-h dietary recalls, possible reverse causation, residual confounding, and exposure misclassification, the clinical and public health implications of these non-linear patterns remain uncertain. Therefore, these findings should be regarded as hypothesis-generating, and no recommendation regarding higher sodium intake or subgroup-specific sodium targets should be inferred from the present analyses.

### 4.5 Strengths and limitations

This study has several strengths. First, we examined table salt addition and depressive symptom dimensions, going beyond total depression scores. This approach helps capture heterogeneity within the depressive spectrum and identify symptom clusters that may be more strongly related to sodium-related exposures. Second, we applied NHANES complex sampling weights to obtain nationally representative estimates and further assessed the consistency of the findings in an independent Chinese clinical cohort. Despite differences in population characteristics, the main patterns were broadly similar across the two cohorts. Third, restricted cubic spline modeling revealed non-linear dose-response relationships between dietary sodium intake and depressive symptoms, allowing a more detailed description of the shape of these associations.

This study also has several limitations. First, the cross-sectional design limits causal inference, and reverse causation cannot be excluded because depressive symptoms may alter appetite, taste preference, eating behavior, and table salt addition. Second, dietary sodium intake and table salt addition were assessed using 24-hour dietary recalls and self-reports, which may introduce recall bias and exposure misclassification compared with 24-h urinary sodium measurements. Third, table salt addition is only a partial proxy for total salt exposure and may not fully capture salt from cooking, processed foods, restaurant foods, sauces, pickled foods, or broader dietary patterns. It may also reflect salt preference, food choice, cultural dietary behavior, or socioeconomic factors rather than sodium intake itself. Fourth, residual confounding cannot be excluded despite multivariable adjustment. Total energy intake, overall diet quality, potassium intake, physical activity, antidepressant use, psychiatric history, and other medication use were not consistently available or could not be reliably harmonized across cohorts. Sleep-related variables were also not adjusted for because sleep disturbance was one of the PHQ-9 symptom outcomes, and additional adjustment for sleep may have introduced overadjustment. Fifth, the nadir values identified in the spline analyses should not be interpreted as recommended, beneficial, safe, or optimal sodium intake levels because they were exploratory estimates derived from cross-sectional data. Finally, the US and Chinese cohorts were derived from different source populations, namely a community-based national sample and a psychiatric clinical sample. The significant baseline differences between cohorts, together with the smaller sample size and sparse events for some symptoms in the Chinese cohort, may have influenced the estimates and limited statistical power. Therefore, the Chinese cohort should be interpreted as an independent clinical comparison cohort used to assess directional consistency rather than as a formal validation or replication cohort, and cross-cohort comparisons should be made cautiously.

## 5 Conclusion

In this cross-population analysis of a US national sample and an independent Chinese clinical cohort, habitual table salt addition was associated with a greater burden of depressive symptoms, particularly somatic symptoms such as fatigue, sleep disturbance, and appetite change. Dietary sodium intake showed non-linear associations with the odds of depressive symptom presence and depressive symptom scores in the two cohorts. However, the nadir values observed in the spline analyses should not be interpreted as optimal, beneficial, safe, or recommended sodium intake targets. Overall, these findings suggest that sodium-related exposures may be associated with depressive symptom profiles across symptom domains, while underscoring the need for prospective and interventional studies to clarify temporality, causality, and clinical relevance.

## Data Availability

The NHANES data are publicly available. The de-identified data from the Gansu cohort supporting the conclusions of this article will be made available by the corresponding authors upon reasonable request, subject to institutional and ethical requirements.

## Supplementary material

Supplementary Table 1 Uncorrected and multiple-testing corrected *P*-values for associations between table salt addition and individual depressive symptoms.

Supplementary Table 2 Uncorrected and multiple-testing corrected *P-*values for associations between table salt addition and depressive symptom dimensions.

Supplementary Table 3 Uncorrected and multiple-testing corrected *P*-values for non-linear associations between dietary sodium intake and depressive symptoms or dimensions.

Supplementary Table 4 Uncorrected and multiple-testing corrected *P*-values for subgroup interaction tests.
