# Supplementary material for: Dietary sodium, table salt use, and specific depressive symptoms: cross-sectional associations in NHANES and an independent Chinese clinical cohort
Source: Front Nutr. 2026 Jun 15;13:1838842. doi: 10.3389/fnut.2026.1838842 (PMC13310660; doi:10.3389/fnut.2026.1838842)
Supplement: Supplementary Table 1 — Uncorrected and multiple-testing corrected P-values for associations between table salt addition and individual depressive symptoms. [file Table_1.docx]

**Supplementary Table S1.** Uncorrected and multiple-testing corrected *P* values for associations between table salt addition and individual depressive symptoms.

| **Cohort** | **Symptom** | **Model** | **Uncorrected *P*** | **Bonferroni-corrected *P*** | **Sidak-corrected *P*** |
| --- | --- | --- | --- | --- | --- |
| NHANES | Anhedonia | Model 1 | <0.001 | <0.001 | <0.001 |
|  |  | Model 2 | <0.001 | 0.002 | 0.002 |
|  |  | Model 3 | 0.001 | 0.012 | 0.012 |
|  | Depressed mood | Model 1 | <0.001 | 0.001 | 0.001 |
|  |  | Model 2 | 0.002 | 0.017 | 0.017 |
|  |  | Model 3 | 0.007 | 0.065 | 0.063 |
|  | Sleep disturbance | Model 1 | <0.001 | <0.001 | <0.001 |
|  |  | Model 2 | <0.001 | <0.001 | <0.001 |
|  |  | Model 3 | <0.001 | 0.006 | 0.006 |
|  | Fatigue | Model 1 | <0.001 | <0.001 | <0.001 |
|  |  | Model 2 | <0.001 | <0.001 | <0.001 |
|  |  | Model 3 | <0.001 | <0.001 | <0.001 |
|  | Appetite change | Model 1 | <0.001 | 0.001 | 0.001 |
|  |  | Model 2 | <0.001 | 0.006 | 0.006 |
|  |  | Model 3 | 0.003 | 0.023 | 0.023 |
|  | Low self-esteem | Model 1 | <0.001 | 0.001 | 0.001 |
|  |  | Model 2 | <0.001 | 0.005 | 0.005 |
|  |  | Model 3 | 0.002 | 0.017 | 0.017 |
|  | Concentration problems | Model 1 | <0.001 | 0.005 | 0.004 |
|  |  | Model 2 | 0.003 | 0.027 | 0.026 |
|  |  | Model 3 | 0.008 | 0.073 | 0.071 |
|  | Psychomotor changes | Model 1 | <0.001 | 0.003 | 0.003 |
|  |  | Model 2 | 0.009 | 0.085 | 0.082 |
|  |  | Model 3 | 0.031 | 0.278 | 0.246 |
|  | Suicidal ideation | Model 1 | 0.064 | 0.575 | 0.448 |
|  |  | Model 2 | 0.190 | 1.000 | 0.850 |
|  |  | Model 3 | 0.207 | 1.000 | 0.877 |
| Gansu Provincial People’s Hospital | Anhedonia | Model 1 | 0.050 | 0.447 | 0.368 |
|  |  | Model 2 | 0.067 | 0.602 | 0.464 |
|  |  | Model 3 | 0.042 | 0.376 | 0.319 |
|  | Depressed mood | Model 1 | 0.006 | 0.050 | 0.049 |
|  |  | Model 2 | 0.007 | 0.062 | 0.060 |
|  |  | Model 3 | 0.011 | 0.099 | 0.095 |
|  | Sleep disturbance | Model 1 | 0.012 | 0.112 | 0.107 |
|  |  | Model 2 | 0.009 | 0.081 | 0.078 |
|  |  | Model 3 | 0.029 | 0.259 | 0.231 |
|  | Fatigue | Model 1 | 0.014 | 0.122 | 0.115 |
|  |  | Model 2 | 0.014 | 0.122 | 0.116 |
|  |  | Model 3 | 0.013 | 0.118 | 0.112 |
|  | Appetite change | Model 1 | 0.025 | 0.222 | 0.201 |
|  |  | Model 2 | 0.013 | 0.120 | 0.114 |
|  |  | Model 3 | 0.018 | 0.161 | 0.150 |
|  | Low self-esteem | Model 1 | 0.147 | 1.000 | 0.761 |
|  |  | Model 2 | 0.105 | 0.942 | 0.630 |
|  |  | Model 3 | 0.149 | 1.000 | 0.766 |
|  | Concentration problems | Model 1 | 0.011 | 0.097 | 0.093 |
|  |  | Model 2 | 0.011 | 0.095 | 0.091 |
|  |  | Model 3 | 0.019 | 0.172 | 0.160 |
|  | Psychomotor changes | Model 1 | / | / | / |
|  |  | Model 2 | / | / | / |
|  |  | Model 3 | / | / | / |
|  | Suicidal ideation | Model 1 | 0.536 | 1.000 | 0.999 |
|  |  | Model 2 | 0.506 | 1.000 | 0.998 |
|  |  | Model 3 | 0.388 | 1.000 | 0.988 |

Model 1: unadjusted.
Model 2: adjusted for age, gender, education, marital status, and PIR.
Model 3: Model 2 + BMI, smoking status, alcohol drinking status, diabetes, hypertension, and CVD.
Bonferroni- and Sidak-corrected *P* values were calculated within each cohort and each model across the prespecified nine PHQ-9 symptom outcomes. Psychomotor changes in the Gansu cohort were excluded because of sparse outcomes and model instability. Corresponding ORs and 95% CIs are presented in Table 2. Abbreviations: PIR, poverty-income ratio; BMI, body mass index; CVD, cardiovascular disease.

Supplementary Table S2. Uncorrected and multiple-testing corrected *P* values for associations between table salt addition and depressive symptom dimensions.

| **Cohort** | **Dimension** | **Model** | **Uncorrected *P*** | **Bonferroni-corrected *P*** | **Sidak-corrected *P*** |
| --- | --- | --- | --- | --- | --- |
| NHANES | Somatic dimension | Model 1 | <0.001 | <0.001 | <0.001 |
|  |  | Model 2 | <0.001 | <0.001 | <0.001 |
|  |  | Model 3 | <0.001 | <0.001 | <0.001 |
|  | Cognitive dimension | Model 1 | <0.001 | <0.001 | <0.001 |
|  |  | Model 2 | <0.001 | <0.001 | <0.001 |
|  |  | Model 3 | <0.001 | <0.001 | <0.001 |
| Gansu Provincial People’s Hospital | Somatic dimension | Model 1 | 0.028 | 0.055 | 0.055 |
|  |  | Model 2 | 0.014 | 0.031 | 0.031 |
|  |  | Model 3 | 0.041 | 0.083 | 0.081 |
|  | Cognitive dimension | Model 1 | 0.215 | 0.429 | 0.383 |
|  |  | Model 2 | 0.148 | 0.296 | 0.274 |
|  |  | Model 3 | 0.192 | 0.383 | 0.347 |

Model 1: unadjusted.

Model 2: adjusted for age, gender, education, marital status, and PIR.

Model 3: Model 2 + BMI, smoking status, alcohol drinking status, diabetes, hypertension, and CVD.

Bonferroni- and Sidak-corrected P values were calculated within each cohort and each model across the two depressive symptom dimensions.

Corresponding β coefficients and 95% CIs are presented in Table 3. Gansu refers to the independent Chinese clinical cohort from Gansu Provincial People’s Hospital. Abbreviations: PIR, poverty-income ratio; BMI, body mass index; CVD, cardiovascular disease.

**Supplementary Table S3.** Uncorrected and multiple-testing corrected *P* values for non-linear associations between dietary sodium intake and depressive symptoms or dimensions.

| **Cohort** | **Outcome type** | **Outcome** | **Uncorrected *P* for non-linearity** | **Bonferroni-corrected *P*** | **Sidak-corrected *P*** |
| --- | --- | --- | --- | --- | --- |
| NHANES | Individual depressive symptom | Anhedonia | 0.177 | 1.000 | 0.827 |
| Gansu Provincial People’s Hospital |  |  | <0.001 | 0.008 | 0.008 |
| NHANES |  | Depressed mood | <0.001 | <0.001 | <0.001 |
| Gansu Provincial People’s Hospital |  |  | 0.003 | 0.023 | 0.023 |
| NHANES |  | Sleep disturbance | <0.001 | <0.001 | <0.001 |
| Gansu Provincial People’s Hospital |  |  | <0.001 | <0.001 | <0.001 |
| NHANES |  | Fatigue | <0.001 | <0.001 | <0.001 |
| Gansu Provincial People’s Hospital |  |  | 0.013 | 0.114 | 0.108 |
| NHANES |  | Appetite change | 0.001 | 0.011 | 0.011 |
| Gansu Provincial People’s Hospital |  |  | <0.001 | <0.001 | <0.001 |
| NHANES |  | Low self-esteem | <0.001 | 0.003 | 0.003 |
| Gansu Provincial People’s Hospital |  |  | 0.074 | 0.667 | 0.500 |
| NHANES |  | Concentration problems | 0.002 | 0.019 | 0.019 |
| Gansu Provincial People’s Hospital |  |  | 0.068 | 0.616 | 0.472 |
| NHANES |  | Psychomotor changes | <0.001 | 0.004 | 0.004 |
| Gansu Provincial People’s Hospital |  |  | 0.280 | 1.000 | 0.948 |
| NHANES |  | Suicidal ideation | 0.063 | 0.569 | 0.444 |
| Gansu Provincial People’s Hospital |  |  | 0.792 | 1.000 | 1.000 |
| NHANES | Depressive symptom dimension | Somatic dimension | <0.001 | <0.001 | <0.001 |
| Gansu Provincial People’s Hospital |  |  | <0.001 | <0.001 | <0.001 |
| NHANES |  | Cognitive dimension | <0.001 | <0.001 | <0.001 |
| Gansu Provincial People’s Hospital |  |  | <0.001 | 0.001 | 0.001 |

Non-linearity was assessed using restricted cubic spline models. Weighted models were used for NHANES, and unweighted models were used for the Gansu cohort. Bonferroni- and Sidak-corrected *P* values were calculated within each cohort and outcome type. Correction was applied across the nine individual depressive symptoms and across the two depressive symptom dimensions separately. Gansu refers to the independent Chinese clinical cohort from Gansu Provincial People’s Hospital. Abbreviations: RCS, restricted cubic spline.

**Supplementary Table S4.** Uncorrected and multiple-testing corrected *P* values for subgroup interaction tests.

| **Cohort** | **Subgroup factor** | **Outcome** | **Uncorrected *P* for interaction** | **Bonferroni-corrected *P* for interaction** | **Sidak-corrected *P* for interaction** |
| --- | --- | --- | --- | --- | --- |
| NHANES | Age group | Anhedonia | 0.423 | 1.000 | 0.993 |
|  |  | Depressed mood | 0.230 | 1.000 | 0.905 |
|  |  | Sleep disturbance | 0.191 | 1.000 | 0.852 |
|  |  | Fatigue | 0.269 | 1.000 | 0.941 |
|  |  | Appetite change | 0.927 | 1.000 | 1.000 |
|  |  | Low self-esteem | 0.911 | 1.000 | 1.000 |
|  |  | Concentration problems | 0.015 | 0.135 | 0.127 |
|  |  | Psychomotor changes | 0.706 | 1.000 | 1.000 |
|  |  | Suicidal ideation | 0.088 | 0.794 | 0.564 |
|  | Gender | Anhedonia | 0.700 | 1.000 | 1.000 |
|  |  | Depressed mood | 0.345 | 1.000 | 0.978 |
|  |  | Sleep disturbance | 0.208 | 1.000 | 0.878 |
|  |  | Fatigue | 0.585 | 1.000 | 1.000 |
|  |  | Appetite change | 0.836 | 1.000 | 1.000 |
|  |  | Low self-esteem | 0.495 | 1.000 | 0.998 |
|  |  | Concentration problems | 0.269 | 1.000 | 0.940 |
|  |  | Psychomotor changes | 0.686 | 1.000 | 1.000 |
|  |  | Suicidal ideation | 0.721 | 1.000 | 1.000 |
|  | Education | Anhedonia | 0.533 | 1.000 | 0.999 |
|  |  | Depressed mood | 0.708 | 1.000 | 1.000 |
|  |  | Sleep disturbance | 0.910 | 1.000 | 1.000 |
|  |  | Fatigue | 0.072 | 0.645 | 0.488 |
|  |  | Appetite change | 0.562 | 1.000 | 0.999 |
|  |  | Low self-esteem | 0.577 | 1.000 | 1.000 |
|  |  | Concentration problems | 0.708 | 1.000 | 1.000 |
|  |  | Psychomotor changes | 0.344 | 1.000 | 0.977 |
|  |  | Suicidal ideation | 0.125 | 1.000 | 0.698 |
|  | Hypertension | Anhedonia | 0.941 | 1.000 | 1.000 |
|  |  | Depressed mood | 0.111 | 1.000 | 0.653 |
|  |  | Sleep disturbance | 0.946 | 1.000 | 1.000 |
|  |  | Fatigue | 0.200 | 1.000 | 0.865 |
|  |  | Appetite change | 0.551 | 1.000 | 0.999 |
|  |  | Low self-esteem | 0.868 | 1.000 | 1.000 |
|  |  | Concentration problems | 0.368 | 1.000 | 0.984 |
|  |  | Psychomotor changes | 0.436 | 1.000 | 0.994 |
|  |  | Suicidal ideation | 0.144 | 1.000 | 0.754 |
| Gansu Provincial People’s Hospital | Age group | Anhedonia | 0.950 | 1.000 | 1.000 |
|  |  | Depressed mood | 0.248 | 1.000 | 0.923 |
|  |  | Sleep disturbance | 0.512 | 1.000 | 0.998 |
|  |  | Fatigue | 0.958 | 1.000 | 1.000 |
|  |  | Appetite change | 0.342 | 1.000 | 0.977 |
|  |  | Low self-esteem | 0.527 | 1.000 | 0.999 |
|  |  | Concentration problems | 0.211 | 1.000 | 0.882 |
|  |  | Psychomotor changes | / | / | / |
|  |  | Suicidal ideation | 1.000 | 1.000 | 1.000 |
|  | Gender | Anhedonia | 0.195 | 1.000 | 0.858 |
|  |  | Depressed mood | 0.387 | 1.000 | 0.988 |
|  |  | Sleep disturbance | 0.074 | 0.666 | 0.499 |
|  |  | Fatigue | 0.534 | 1.000 | 0.999 |
|  |  | Appetite change | 0.101 | 0.911 | 0.617 |
|  |  | Low self-esteem | 0.342 | 1.000 | 0.977 |
|  |  | Concentration problems | 0.744 | 1.000 | 1.000 |
|  |  | Psychomotor changes | / | / | / |
|  |  | Suicidal ideation | 0.398 | 1.000 | 0.990 |
|  | Education | Anhedonia | 0.456 | 1.000 | 0.996 |
|  |  | Depressed mood | 0.403 | 1.000 | 0.990 |
|  |  | Sleep disturbance | 0.427 | 1.000 | 0.993 |
|  |  | Fatigue | 0.227 | 1.000 | 0.902 |
|  |  | Appetite change | 0.911 | 1.000 | 1.000 |
|  |  | Low self-esteem | 0.573 | 1.000 | 1.000 |
|  |  | Concentration problems | 0.208 | 1.000 | 0.878 |
|  |  | Psychomotor changes | / | / | / |
|  |  | Suicidal ideation | 0.127 | 1.000 | 0.706 |
|  | Hypertension | Anhedonia | 0.094 | 0.848 | 0.590 |
|  |  | Depressed mood | 0.435 | 1.000 | 0.994 |
|  |  | Sleep disturbance | 0.341 | 1.000 | 0.976 |
|  |  | Fatigue | 0.956 | 1.000 | 1.000 |
|  |  | Appetite change | 0.705 | 1.000 | 1.000 |
|  |  | Low self-esteem | 0.560 | 1.000 | 0.999 |
|  |  | Concentration problems | 0.050 | 0.447 | 0.368 |
|  |  | Psychomotor changes | / | / | / |
|  |  | Suicidal ideation | 0.354 | 1.000 | 0.981 |

Subgroup interaction analyses were conducted in both the NHANES cohort and the independent Chinese clinical cohort from Gansu Provincial People’s Hospital. Interaction *P* values were obtained from adjusted logistic regression models including table salt addition, each subgroup factor, their interaction term, and covariates. NHANES analyses used survey-weighted logistic regression incorporating sampling weights, strata, and primary sampling units; Gansu cohort analyses used unweighted logistic regression. Bonferroni- and Sidak-corrected *P* values were calculated within each cohort and subgroup factor across the prespecified nine PHQ-9 symptom outcomes using unrounded P values. Psychomotor changes in the Gansu cohort were not tested because of sparse outcomes and model instability, and are indicated by “/”. Abbreviation: PHQ-9, Patient Health Questionnaire-9.
